# Metabolomics analysis of pathways underlying radiation-induced salivary gland dysfunction stages

**DOI:** 10.1371/journal.pone.0294355

**Published:** 2023-11-20

**Authors:** Lauren G. Buss, Diogo De Oliveira Pessoa, Justin M. Snider, Megha Padi, Jessica A. Martinez, Kirsten H. Limesand

**Affiliations:** 1 School of Nutritional Sciences and Wellness, University of Arizona, Tucson, AZ, United States of America; 2 Biostatistics and Bioinformatics Shared Resource, Arizona Cancer Center, University of Arizona, Tucson, AZ, United States of America; 3 University of Arizona Cancer Center, Tucson, AZ, United States of America; 4 Department of Molecular and Cellular Biology, University of Arizona, Tucson, AZ, United States of America; University of the Philippines Diliman, PHILIPPINES

## Abstract

Salivary gland hypofunction is an adverse side effect associated with radiotherapy for head and neck cancer patients. This study delineated metabolic changes at acute, intermediate, and chronic radiation damage response stages in mouse salivary glands following a single 5 Gy dose. Ultra-high performance liquid chromatography-mass spectrometry was performed on parotid salivary gland tissue collected at 3, 14, and 30 days following radiation (IR). Pathway enrichment analysis, network analysis based on metabolite structural similarity, and network analysis based on metabolite abundance correlations were used to incorporate both metabolite levels and structural annotation. The greatest number of enriched pathways are observed at 3 days and the lowest at 30 days following radiation. Amino acid metabolism pathways, glutathione metabolism, and central carbon metabolism in cancer are enriched at all radiation time points across different analytical methods. This study suggests that glutathione and central carbon metabolism in cancer may be important pathways in the unresolved effect of radiation treatment.

## Introduction

Over 54,000 new cases of head and neck cancer (HNC) are estimated in the United States by the American Cancer Society annually, with approximately 5% of these cases resulting in death [1]. Radiotherapy, combined with chemotherapy and surgery, is the dominant treatment for HNC and is effective in eliminating tumors and preventing cancer recurrence [2, 3]. Due to the proximity of the salivary glands to HNC tumors, they are indirectly damaged by radiotherapy and lose secretory function due to their high level of radiation sensitivity [4–6]. Acutely, oral mucositis and loss of saliva production begins to occur within the first week of radiotherapy as loss of acinar cells and glandular shrinkage occurs [6, 7]. Hyposalivation continues to persist chronically in over 80% of HNC patients [8–10]. Failure of the acinar cells to functionally repair and regenerate contributes to chronic salivary gland dysfunction, with the level of acute damage reflecting the level of chronic complications, such as dental caries, periodontitis, and nutritional deficiencies [11–14]. The underlying mechanisms regulating chronic radiation-induced salivary gland dysfunction are not well understood [6]. Current treatments to temporarily relieve symptoms include sialagogues without restoring function to the damaged salivary gland, and are associated with unpleasant side effects and inconsistent results [15, 16]. Therefore, the underlying mechanisms of radiation-induced salivary gland damage need to be discovered to identify methods for restoring function to the damaged gland.

Metabolomics is a powerful platform that reflects the effects of disease or damage in cells, tissues, or biological fluids since the metabolic phenotype is the interaction of multiple environmental factors in combination with genetic factors [17–20]. Small changes in gene expression or protein levels are magnified at the metabolite level, which suggests that changes in metabolite levels may provide a clearer reflection of an organism’s phenotype compared to changes at the gene and protein level [21]. Metabolomics has been used to identify mechanisms underlying the radiation-damage response in multiple tissue types. The effects of different radiation dosages (0, 2, and 20 Gy) on the intestinal tissue metabolite profile in C57BL/6 mice revealed increases in amino acid levels that correlated with increased radiation dosage, which the authors suggest might be reflective of oxidative stress and pose amino acid metabolism as a possible therapeutic target mechanism to alleviate radiation-induced intestinal toxicity [22]. The effect of 12 Gy partial body radiation on liver, kidney, heart, lung, and small intestinal tissue of non-human primates at acute and intermediate damage time points revealed temporal changes in both citrulline and branched chain amino acids in all tissue types [23], which may underlie chronic damage.

Our previous work integrated metabolites and transcripts altered in response to radiation treatment at five days post-IR in mouse parotid salivary gland tissue and identified joint pathway enrichment for glutathione metabolism, energy metabolism (TCA cycle and thermogenesis), bile acid production, and peroxisomal lipid metabolism [24]. Decreases in apical/basolateral polarity and increases in compensatory proliferation in the acinar cell compartment have been demonstrated at five days post-IR, with continued increases in compensatory proliferation correlated with chronic loss of secretory function [25, 26]. Since loss of secretory function has been previously demonstrated to begin as early as three days post-IR and continue for at least 90 days to one year in rodent models, these data suggest that the aforementioned metabolic pathways may drive chronic salivary gland dysfunction [24–28].

**Fig 1:**
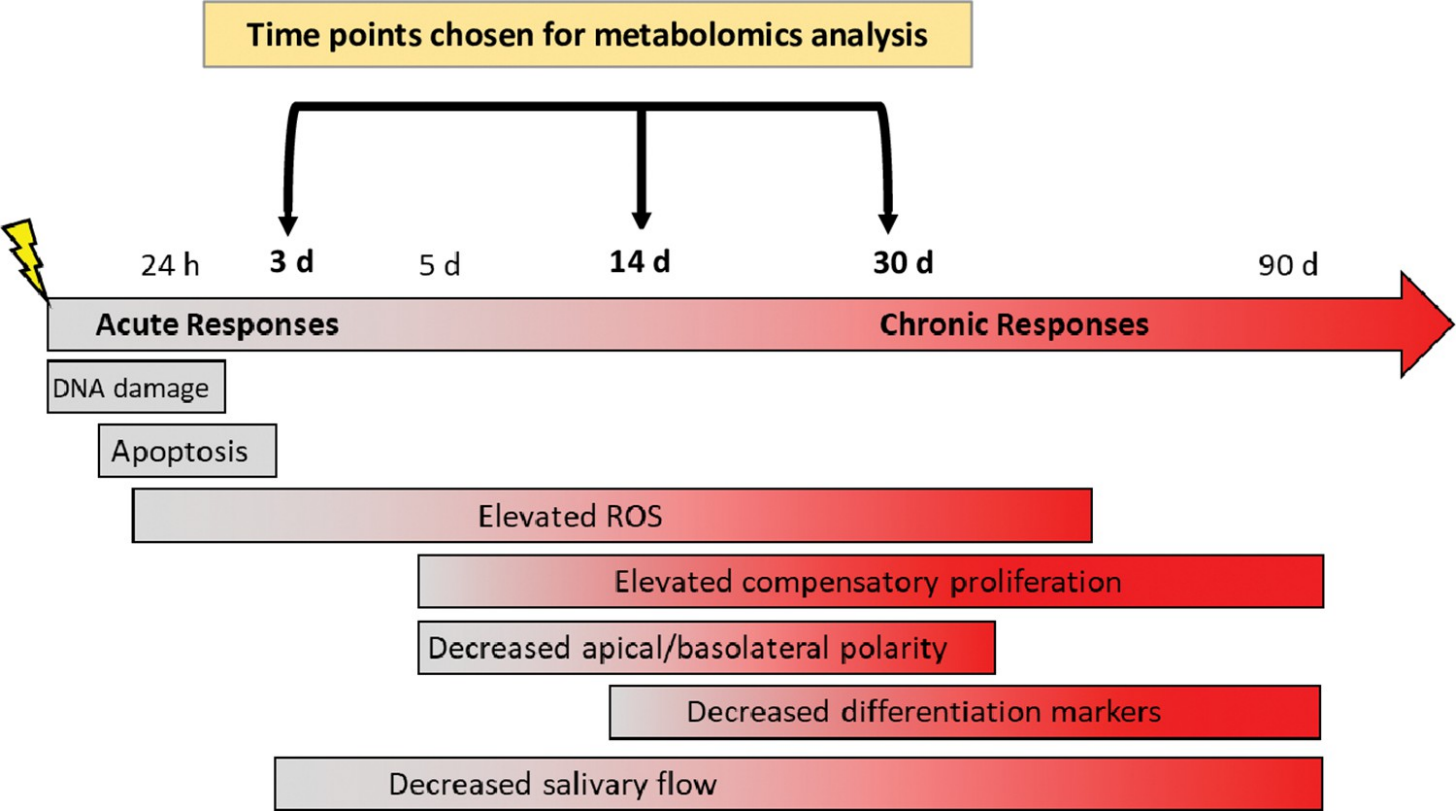
Summary of previously identified phenotypes underlying the radiation damage response in the murine salivary gland over time following exposure to a single 5 Gy dose. Abbreviations: h = hours, d = days, DNA = deoxyribonucleic acid, ROS = reactive oxygen species.

The purpose of this study is to identify metabolic changes that occur at acute, intermediate, and chronic damage time points in the salivary gland due to radiation treatment. A summary of previously identified phenotypes underlying the radiation-damage response over time in a mouse model is presented for context (Fig 1). Apoptosis of salivary acinar cells has been reported between 8–72 hours following radiation [29, 30] and increased levels of reactive oxygen species (ROS) have been detected as early as 24 hours post-IR and persist for at least ten days [31]. Loss of secretory function has been reported as early as three days post-IR [27, 32], therefore, three days post-IR was chosen as a representative acute damage time point. Decreases in acinar cell differentiation markers (e.g. amylase) have been reported as early as ten days post-IR and persist for at least 90 days post-treatment [27, 33–35]. Fourteen days post-IR was selected as a representative intermediate damage time point as it exhibits increased compensatory proliferation, decreased differentiation and decreased function and we hypothesize may be a pivotal time point in the wound healing response [36]. We have previously demonstrated that radiation-induced loss of secretory function at 30 days post-treatment is similar at 60 and 90 day post-treatment [27], and other rodent models have confirmed chronic loss of function up to one year post-treatment [28, 37]. Therefore 30 days was chosen as a representative chronic damage time point as it exhibits increased compensatory proliferation, decreased differentiation and decreased function. To increase the robustness of the analysis, we are using metabolite annotation, metabolite structure, and metabolite levels to identify metabolic pathway-level enrichment. We articulated the metabolic changes that are correlated with the different stages of the radiation-induced damage response in the salivary gland and placed them in a biological context through network analysis.

## Methods

### Mice and radiation treatment

Mice were housed and treated following protocols approved by the University of Arizona Institutional Animal Care and Use Committee (IACUC). All experiments were conducted using female FVB mice obtained from Jackson Laboratories (Bar Harbor, ME). At 4–6 weeks of age, mice were treated with a single five Gy radiation dose using a ^60^Cobalt Teletherapy Instrument from Atomic Energy of Canada Ltd Theratron (80-cm distance from source). Prior to radiation treatment, mice were anesthetized with an intraperitoneal injection of ketamine/xylazine (70mg/kg-10 mg/mL) and placed in a 50 mL conical tube. To target the head and neck region for radiation treatment, the rest of the body was shielded with >6mm thick lead during radiation exposure.

### Tissue preparation and metabolomics processing

Parotid salivary glands were extracted from mice at 3 days post-IR (N = 8), 14 days post-IR (N = 8), 30 days post-IR (N = 8), and from untreated mice (N = 4). The sample size of the untreated group decreased from 8 to 4 due to severe dehydration occurring in 4 of the mice due to a failure in the automatic water system. 10–20 mg of parotid salivary gland tissue sample was subjected to a methanol (1 mL) extraction of both polar and nonpolar metabolites. Samples were homogenized in a MPbio FastPrep 24 bead beater utilizing 2 mL homogenization tubes, Glass beads (Sigma 425–600 μm acid washed, G8772-100 G), and 1 mL methanol spiked with internal standard mix (10 μL Deuterated Amino Mix, 10 μL of SPLASH LipidoMIX Internal standard mixture, Avanti, Al; Product number 330707) for additional semiquantitative analysis. Sample were homogenized under the following Fast prep conditions: twice at 6.5 m/s for 20 Sec. Tissue precipitant was pelleted through centrifugation (10 min, 10,000 RPM, 4°C), supernatant was transferred and dried under nitrogen and further stored at -20°C until resuspended in 100 μL methanol/0.1% formic acid.

### Ultra-high performance liquid chromatography-mass spectrometry (UHPLC-MS) analysis

One μL of sample extract was injected onto a Thermo Vanquish Duo UHPLC system in randomized order and separated using a Thermo Scientific Accucore 150-Amide-HILIC (250 x 2.1 mm 2.6 μ) and Hypersil GOLD (150 x 2.1mm 1.9μ) columns for hydrophilic interaction liquid chromatography (HILIC) and reverse phase (RP) chromatography, respectively, as described by Najdekr et al. [38]. The HILIC solvent system included a gradient from solvent A (95% acetonitrile/ 5% water with 10 mM ammonium acetate and 0.1% formic acid) to solvent B (50% acetonitrile/ 50% water with 10 mM ammonium acetate and 0.1% acetic acid) over 12 mins at 500 μL/min, and column re-equilibration occurs during RP analysis. The RP chromatography included a gradient from solvent A (0.1% formic acid in water) to solvent B (0.1% formic acid in methanol) over 12 minutes at 300 μL/min, and column re-equilibration occurs during HILIC analysis. Column temperatures were maintained at 50°C. Mass spectrometry (MS) detection was performed using a state-of-the-art Thermo Exploris 480, utilizing default lipidomic and metabolomic acquisition settings optimized by Thermo unless otherwise stated. These settings include: 3.4kV spray voltage in positive ion mode, 45 AU sheath gas, 10 AU auxiliary gas, and 325°C ion transfer tube, 350°C vaporizer temperature. The Orbitrap mass analyzer scanned from 67–1000 m/z at 120,000 resolution for full scans and 15,000 for MS/MS scans. Samples were run in MS mode only, while Pooled QC’s were run in data-dependent acquisition (20 scans) mode and dynamic exclusion was set to 8 sec utilizing higher-energy collisional dissociation (HCD). Thermo AquireX platform was utilized on the pooled QC sample to achieve optimal feature annotation.

### Metabolite ID annotation

Compound Discoverer version 3.3 provided Compound Names, SMILES IDs and KEGG IDs for a portion of the detected metabolites as part of its output. A single Pooled QC was created as a composited from all sample groups (aliquots were combined post extraction) and run between every 15 samples during LC/MS analysis, then utilized post processing to remove features with greater that 30% variation in the QC samples. The QC sample was also utilized for peak area and retention time normalization over the entire run. Annotations were assigned in Compound Discoverer utilizing the following data bases: MZcloud, Metabolika, ChemSpider, and MassList. Features with predicted formulas (only) based on accurate mass were also exported as part of the output. PubChem ID annotation was carried out by querying the PubChem database primarily from compound names and secondarily from compound formulas using the function *get_cid* from the *webchem* package. KEGG IDs that were not supplied by Compound Discoverer were annotated primarily by querying the Chemical Translation Service (CTS) database from compound names using the *cts_convert* function from the *webchem* package and secondarily by querying the KEGG database from compound formulas using the *keggFind* function from the *KEGGREST* package. SMILES IDs that were not supplied by compound discoverer were annotated by querying the PubChem database from PubChem IDs using the *pc_prop* function from the *webchem* package. In HILIC mode, 1,540 compounds were detected and 598 metabolites (39%) were annotated at each timepoint. In RP mode, 2,852 compounds were detected and 1,172 metabolites (30%) were annotated at each time point.

### Metabolite class annotation

Metabolite class annotation was performed using the HMDB database (HMDB Version 5.0) first, and if the compound was not identified in HMDB then the PubChem database (National Library of Medicine) was used. The compound names were entered into the metabolite search engine and when the correct metabolite was identified, the class information was found under “Chemical Taxonomy” in the HMDB database or under the description of the metabolite in the PubChem database. If the metabolite could not be identified, “NA” was recorded.

### Metabolomics statistical analysis

HILIC and RP peak files were obtained through the Compound Discoverer software version 3.3 (CD3.3). Metabolite intensity counts were loaded with the *Read*.*TextData* function. Data quality assessment was carried out first with the *SanityCheckData* function to evaluate the accuracy of sample and class labels and data structure, and to identify non-numeric values and groups with a variance of 0. RSD’s of the QC samples were calculated and metabolite features with greater than a RSD of 30% were not included. CD 3.3 utilized gap filling node to identify features that were not identified in the first pass, then applied random forrest to impute missing values by following compound profile patterns. Data was normalized with the *Normalization* function (parameters: rowNorm = "NULL", transNorm = "LogNorm", scaleNorm = "ParetoNorm", ratio = FALSE, ratioNum = 20) to perform row-wise normalization, log transformation, and pareto scaling of the metabolomics data. All metabolite data processing steps were carried out using the *MetaboAnalystR* package. Principal components for partial least squares-discriminant analysis (PLS-DA) were provided with the *opls* function from the ropls package. Normalized metabolite data distribution was visualized with PSL-DA using the *ggplot2* package.

### Pathway enrichment analysis

Metabolite set enrichment analysis was carried out with *fora* (over representation analysis) and *fgsea* (pre-ranked set enrichment analysis) functions from the *fgsea* package (parameters: minimal metabolite set size 5, maximal metabolite set size 500). Pathway enrichment was performed against KEGG pathways from the ConsensusPathDB (CPDB) database for metabolites.

### MetaMapp network analysis

Metabolite PubChem and SMILES IDs were annotated primarily from compound names and secondarily from compound formulas. The metabolite annotation was used as input to obtain the structural data file (sdf) information and compute the metabolite structural similarity network, using the Metamapp R package (https://github.com/barupal/metamapp). The generated network files (sif) were used to detect communities with the Louvain algorithm, and overrepresentation analysis (ORA) was applied to each detected cluster with more than 50 metabolites to identify enriched pathways. Enrichment analysis was carried out with the *fora* function from the *fgsea* package (parameters: minimal metabolite set size 5, maximal metabolite set size 500) using all KEGG pathways from the ConsensusPathDB (CPDB) database for metabolites.

### Weighted correlation network analysis (WGCNA)

Normalized metabolite levels were used for assessing correlation patterns using weighted correlation analysis (WGCNA). Briefly, the scale free topology fit R^2^ was computed for a range of values of the soft threshold power (*β*). We applied an R^2^ threshold cutoff of 0.9 to obtain the minimum corresponding soft threshold power and used this value of *β* to calculate the adjacency matrix and subsequent topological overlap matrix. The corresponding dissimilarity matrix (1—TOM) values were hierarchically clustered, and modules were detected using the criteria of a tree height of 0.995 and a minimum of 50 metabolites. Pathway enrichment overrepresentation analysis (ORA) was applied to each detected module, using the *fora* function from the *fgsea* package and CPDB KEGG pathways as described above.

## Results

### Metabolomic profiling reveals separation between all radiation time points

**Fig 2:**
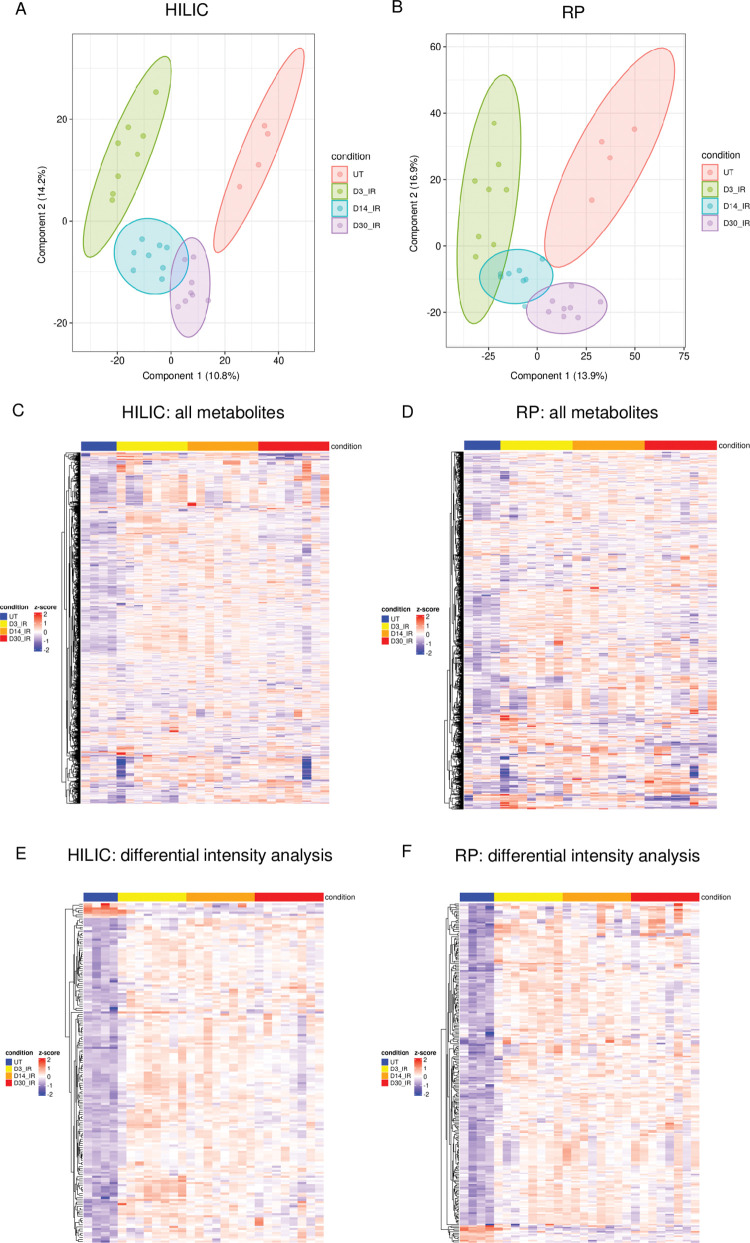
Metabolomic profiling of irradiated parotid salivary glands at days 3 (N = 8), 14 (N = 8), and 30 (N = 8) compared to untreated (N = 4) reveals separation. A) PLS-DA plot of HILIC phase data. B) PLS-DA plot of RP phase data. C) Heatmap of all metabolites identified in HILIC phase. D) Heatmap of all metabolites identified in RP phase. E) Heatmap of differentially altered metabolites in HILIC phase (*P*_adj_<0.05). F) Heatmap of differentially altered metabolites in RP phase (*P*_adj_<0.05). Abbreviations: IR = radiation, HILIC = hydrophilic interaction liquid chromatography, RP = reverse phase chromatography.

The IR time points chosen for this study reflect the acute, intermediate, and chronic damage responses in the salivary gland to identify kinetic changes in metabolites. Selection of radiation dose (5 Gy) was based on consideration of the clinical exposure in patients (2 Gy/day), comparison to other papers in the field (10–40 Gy), and data from our previous studies (2–10 Gy) [39–44]. Metabolomic profiles in each phase were compared between treated and untreated groups at days 3, 14 and 30 post-IR to determine differences at each IR time point. HILIC chromatography was used to separate hydrophilic metabolites while RP chromatography was used to separate polar and aromatic metabolites as well as organic acids, thus improving the scope of metabolites detected [38]. The number of detected features in HILIC phase was 4,600 and the number of detected features in RP phase was 8,525. There were 1,534 metabolites identified by Compound Discoverer in HILIC phase at day 3 IR, 1,531 at day 14 IR, and 1,535 at day 30 IR (S1 Table). There were 2,844 metabolites identified in RP phase at day 3 IR, 2,840 at day 14 IR, and 2,841 at day 30 IR (S1 Table). The partial least squares discriminant analysis reveals distinct metabolite profile separation patterns between the three IR time point groups and the untreated group in both HILIC and RP phases (Fig 2A and 2B). Cross-validation was used to assess the performance and generalizability of the models for both HILIC (S1 Fig) and RP (S2 Fig) datasets. The overall assessment of the model’s performance was good and not overfitted, as the R2 approached 1 for both HILIC and RP, while the Q2 exceeded 0.7 in both models. The heatmaps of all identified metabolites at each time point in HILIC and RP phases display a pattern of increased metabolite levels for each IR group compared to untreated (Fig 2C and 2D). Differential intensity analysis identifies 507 metabolites in HILIC phase and 976 in RP phase with *P*_adj_ < 0.05 between the day 3 IR group and the untreated group, 407 metabolites in HILIC phase and 901 metabolites in RP phase between the day 14 IR group and the untreated group, and 276 metabolites in HILIC phase and 504 metabolites in RP phase between the day 30 IR group and the untreated group (S1 Table). The significant differentially altered metabolites with *P*_adj_ < 0.05 are shown in the heatmaps for each IR group compared to untreated in HILIC and RP phases (Fig 2E and 2F).

We next annotated the top 100 significant metabolites by class for each IR time point (S2 Table). Table 1 presents a summary of the most commonly observed metabolite classes for all IR time points compared to untreated in HILIC and RP phases. We observe across all IR time points that the majority of significant metabolites (*P*_adj_ < 0.01) are upregulated, with the highest percentage of upregulated metabolites observed at day 14 IR and the lowest percentage of upregulated metabolites observed at day 30 IR (Table 1 and S2 Table). Across the 3 IR time points and 2 detection phases, alpha amino acids, carboxylic acids, peptides, and alcohol derivatives are upregulated in response to IR, while most of the significant downregulated metabolites are unidentified, with the few identified downregulated metabolites annotated to amino sugars and organonitrogen compounds (Table 1).

**Table 1:** Summary of the top 100 significant metabolites (*P*_adj_<0.05) annotated with metabolite class information at days 3, 14, and 30 IR compared to untreated in HILIC and RP phases.

IR Time Point	HILIC: number of significant metabolites (*P*adj<0.01)	HILIC: significant upregulated metabolites by class	HILIC: significant downregulated metabolites by class	RP: number of significant metabolites (*P*adj<0.01)	RP: significant upregulated metabolites by class	RP: significant downregulated metabolites by class
Day 3 IR	315 (94% upregulated, 6% downregulated)	alpha amino acids, peptides, azoles, carboxylic acids, 1,2-amino alcohols	unidentified, amino sugars, biotin and derivatives	615 (92% upregulated, 8% downregulated)	prenol lipids, benzene and derivatives, carboxylic acids, diazines, peptides	unidentified, organonitrogen compounds, diazines, phosphocholines
Day 14 IR	271 (96% upregulated, 4% downregulated)	alkaloids, alpha amino acids, n-acylethanolamines, phosphosphingolipids, carboxylic acids, secondary alcohols	unidentified, amino sugars, amines	503 (96% upregulated, 4% downregulated)	fatty acyls, carboxylic acids, piperidines, benzene and derivatives, peptides and dipeptides	unidentified, steroids and derivatives, organonitrogen compounds, diazines
Day 30 IR	139 (91% upregulated, 9% downregulated)	azoles, alpha amino acids, fatty amides, 1,2-amino alcohols, phosphocholines, alkaloids	unidentified, amino sugars, guanines, phosphocholines, pyridines and derivatives	229 (79% upregulated, 21% downregulated)	quinolines, piperidines, benzene and derivatives, fatty acyls, carboxylic acids	unidentified, organonitrogen compounds, diazines, steroids and derivatives, phosphocholines

Abbreviations: IR = radiation, HILIC = hydrophilic interaction liquid chromatography, RP = reverse phase chromatography, *P*adj = a Benjamini Hochberg adjusted *P*-value.

### Pathway enrichment analysis identifies significant enrichment at days 3 and 14 following radiation

We performed enrichment analysis on the metabolites with associated KEGG IDs using gene set enrichment analysis (GSEA) with KEGG pathways and the top 10 enriched pathways at each IR time point are presented in Table 2. We observe 16 significant pathways using *P*_adj_ < 0.25 at day 3 IR in HILIC phase and 3 significant pathways in RP phase (full set of enriched pathways is presented in S3 Table). The top 3 enriched pathways in day 3 IR HILIC phase are glutathione metabolism, aminoacyl-tRNA biosynthesis, and protein digestion and absorption, and the 3 significant pathways in RP phase are cysteine and methionine metabolism, central carbon metabolism in cancer, and glutathione metabolism (Table 2). At day 14 IR, 11 pathways are significantly enriched in HILIC phase and 10 pathways are significantly enriched in RP phase (S3 Table). The top 3 enriched pathways in day 14 IR HILIC phase are ferroptosis, aminoacyl-tRNA biosynthesis, and protein digestion and absorption, and the top 3 enriched pathways in RP phase are central carbon metabolism in cancer, phenylalanine metabolism, and aminoacyl-tRNA biosynthesis (Table 2). At day 30 IR, no pathways reached significance at *P*_adj_ < 0.25 in HILIC phase, but the top enriched pathways are ferroptosis, diabetic cardiomyopathy, and glutathione metabolism (S3 Table). In RP phase at day 30 IR, no pathways reached significance at *P*_adj_ < 0.25, with the top listed pathways being tryptophan metabolism, glutathione metabolism, and ubiquinone and other terpenoid-quinone biosynthesis (Table 2). The leading edge metabolites within the enriched pathways observed at each IR time point compared to untreated are shown in S4 Table. The leading edge metabolites are the metabolites with the highest contribution to the enrichment signal for the enriched pathway, which is comparable to leading edge genes in gene set enrichment analysis (GSEA) [45].

**Table 2:** Gene set enrichment analysis (GSEA) of top 3 pathways enriched at days 3, 14, and 30 IR compared to untreated in HILIC and RP phases using (*P*_adj_<0.25) for statistical significance.

Day 3 IR—HILIC						Day 3 IR—RP					
Pathway	*P*val	*P*adj	log2err	ES	NES	Pathway	*P*val	*P*adj	log2err	ES	NES
Glutathione metabolism—Homo sapiens (human)	0.001	0.013	0.139	0.792	1.648	Cysteine and methionine metabolism—Homo sapiens (human)	0.008	0.222	0.052	0.698	1.523
Aminoacyl-tRNA biosynthesis—Homo sapiens (human)	0.001	0.013	0.129	0.735	1.607	Central carbon metabolism in cancer—Homo sapiens (human)	0.011	0.221	0.044	0.686	1.497
Protein digestion and absorption—Homo sapiens (human)	0.001	0.013	0.129	0.735	1.607	Glutathione metabolism—Homo sapiens (human)	0.013	0.222	0.043	0.793	1.524
**Day 14 IR—HILIC**						**Day 14 IR—RP**					
**Pathway**	***P*val**	***P*adj**	**log2err**	**ES**	**NES**	**Pathway**	***P*val**	***P*adj**	**log2err**	**ES**	**NES**
Ferroptosis—Homo sapiens (human)	0.002	0.022	0.113	0.799	1.665	Central carbon metabolism in cancer—Homo sapiens (human)	0.001	0.025	0.171	0.754	1.640
Aminoacyl-tRNA biosynthesis—Homo sapiens (human)	0.002	0.022	0.100	0.708	1.624	Phenylalanine metabolism—Homo sapiens (human)	0.002	0.025	0.117	0.800	1.638
Protein digestion and absorption—Homo sapiens (human)	0.002	0.022	0.100	0.708	1.624	Aminoacyl-tRNA biosynthesis—Homo sapiens (human)	0.002	0.025	0.105	0.730	1.589

No significant enrichment was observed at day 30 IR. Abbreviations: IR = radiation, HILIC = hydrophilic interaction liquid chromatography, RP = reverse phase chromatography, *P*val = an enrichment *P*-value, *P*adj = a Benjamini Hochberg adjusted *P*-value, log2err = the expected error for the standard deviation of the *P*-value logarithm, ES = enrichment score, NES = enrichment score normalized to mean enrichment of random samples of the same size.

### MetaMapp network analysis identifies groups of structurally similar metabolites altered following radiation

**Fig 3:**
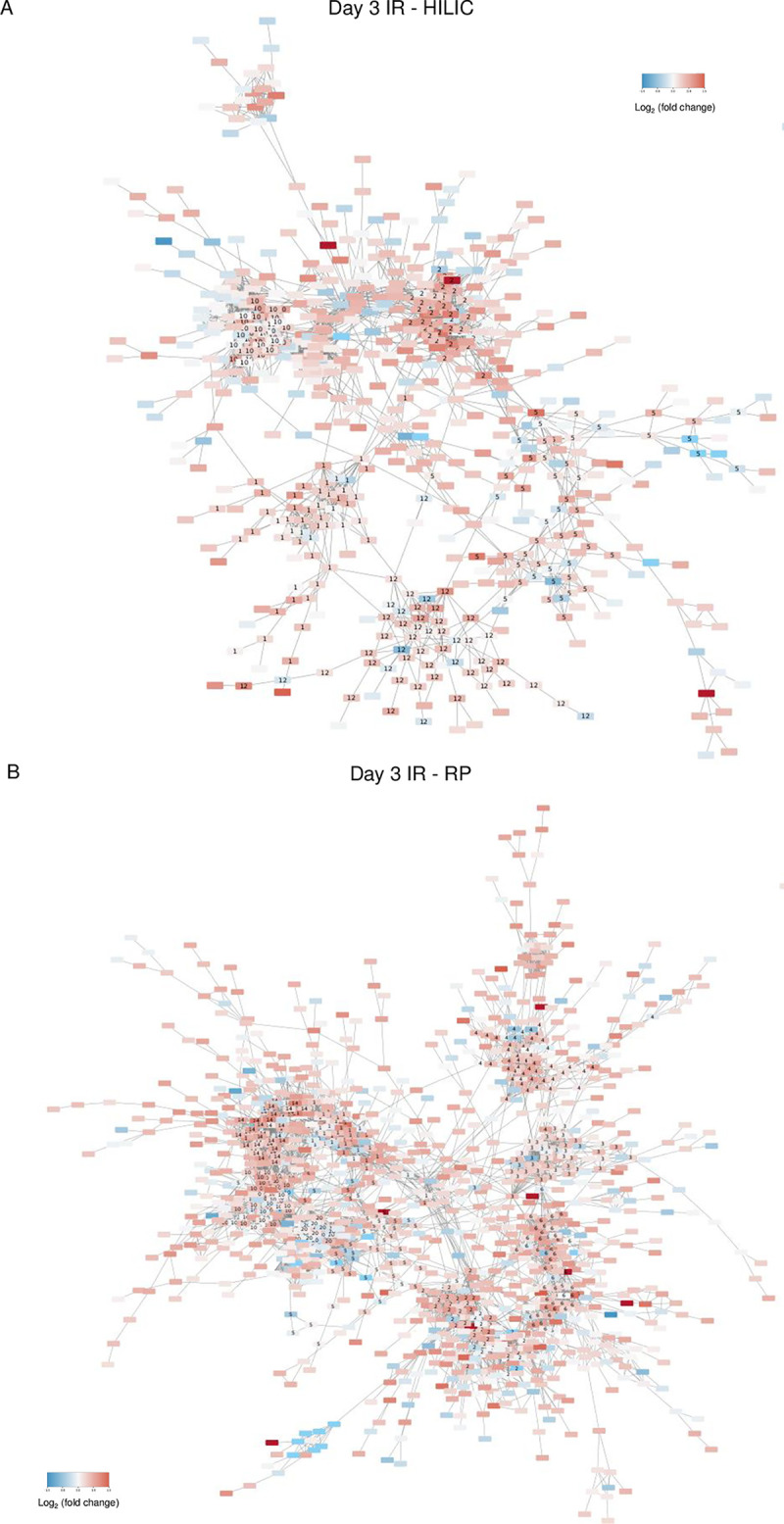
MetaMapp network analysis identifies significant metabolite clusters at day 3 IR. A) HILIC phase. B) RP phase. Rectangles correspond to individual metabolites, edges denote chemical reactions between the metabolites, and numbers denote the communities that clusters of metabolites belong to. Color indicates Log_2_ (fold difference)—red denotes upregulated metabolite levels versus untreated and blue denotes downregulated metabolite levels versus untreated. Abbreviations: IR = radiation, HILIC = hydrophilic interaction liquid chromatography, RP = reverse phase chromatography.

To further investigate metabolite interactions in the context of biological reactions, we created a network of the metabolites based on structural annotation information and subsequently integrated metabolite level information to identify significantly altered modules. MetaMapp creates networks based on chemical and biochemical similarity between metabolites, thus providing improved functional characterization of all metabolites, not just the small fraction that have KEGG IDs [46]. Fig 3A (HILIC) and 3B (RP) illustrate MetaMapp networks from day 3 IR (see S3 Fig for day 14 IR MetaMapp networks and S4 Fig for day 30 IR MetaMapp networks). At day 3 IR HILIC phase, we observe a cluster of 120 metabolites with 42 of them being differentially altered (identified as Community 2 in the MetaMapp network), annotated to arginine biosynthesis as the top pathway (Fig 3A). Communities are defined as clusters of metabolites detected using the Louvain method, and significant communities are defined as those that contain more differentially altered metabolites than expected by chance [47]. Most of the metabolites annotated to Community 2 are upregulated at day 3 IR compared to untreated. This is the only significant community using a hypergeometric p-value cutoff of 0.05 at this time point in HILIC phase (see S5 Table for full list of identified metabolite communities, see S6 Table for full list of pathways annotated to significant metabolite communities). At day 14 IR arginine biosynthesis remains the only significant pathway. At day 30 IR, however, Community 1, consisting of 65 metabolites (25 are differentially altered) is annotated to purine metabolism as the top pathway (Table 3 and S6 Table). In summary, for HILIC phase network analysis across the 3 IR time points, arginine biosynthesis is enriched in the significant metabolite community at day 3 and day 14 IR but not at day 30 IR, whereas lysine degradation and purine metabolism are enriched in significant metabolite communities at day 30 IR.

**Table 3:** Significant (Hypergeometric *P*-value<0.05) MetaMapp metabolite communities at days 3, 14, and 30 IR compared to untreated in HILIC and RP phases.

IR Time Point	Community	Cluster Size	Number of Significant Metabolites	Pathway	Pathway *P*-value	Hypergeometic *P*-value
HILIC Day 3 IR	2	120	42	Arginine biosynthesis—Homo sapiens (human)	2.97E-05	2.16E-08
HILIC Day 14 IR	2	120	33	Arginine biosynthesis—Homo sapiens (human)	2.97E-05	0.0017
HILIC Day 30 IR	2	104	31	Lysine degradation—Homo sapiens (human)	0.0004	7.39E-05
HILIC Day 30 IR	1	65	25	Purine metabolism—Homo sapiens (human)	1.72E-07	0.0212
RP Day 3 IR	14	124	31	Alanine, aspartate and glutamate metabolism—Homo sapiens (human)	1	0.0240
RP Day 3 IR	6	138	30	Phenylalanine metabolism—Homo sapiens (human)	0.0236	0.0459
RP Day 14 IR	14	116	31	Alanine, aspartate and glutamate metabolism—Homo sapiens (human)	1	0.0060
RP Day 14 IR	2	182	30	Tyrosine metabolism—Homo sapiens (human)	0.0002	0.0131
RP Day 30 IR	5	123	30	Tryptophan metabolism—Homo sapiens (human)	6.98E-05	4.53E-05
RP Day 30 IR	9	50	25	Diabetic cardiomyopathy—Homo sapiens (human)	0.0672	0.0068
RP Day 30 IR	2	181	24	Tyrosine metabolism—Homo sapiens (human)	0.0002	0.0147

Abbreviations: IR = radiation, HILIC = hydrophilic interaction liquid chromatography, RP = reverse phase chromatography.

For the RP phase, we observe two significant communities identified as Communities 6 and 14 at day 3 IR (Fig 3B). Community 6 is a cluster of 138 metabolites with 30 significant metabolites and it is enriched in phenylalanine metabolism; Community 14 is a cluster of 124 metabolites with 31 significant metabolites, and is enriched for alanine, aspartate, and glutamate metabolism (Table 3 and S6 Table). Again, most of the significant metabolites are upregulated at day 3 IR compared to untreated. At day 14 IR RP phase, Community 14 remains significant, but not Community 6. Community 2, however, is detected as significant, consists of 182 metabolites (30 are significant) and is enriched for tyrosine metabolism as the top annotated pathway (Table 3 and S6 Table). Community 2 remains significant at day 30 IR RP phase. Additionally, Communities 5 and 9 are significant (Table 3). Community 5 consists of 123 metabolites (30 are significant) and is enriched for tryptophan metabolism. Community 9 consists of 50 metabolites (25 are significant) and is enriched for diabetic cardiomyopathy (Table 3). In summary for RP phase network analysis across the 3 IR time points, the community enriched for alanine, aspartate, and glutamate metabolism is significant at day 3 and day 14 IR but not at day 30 IR. The communities enriched for tryptophan metabolism, diabetic cardiomyopathy, and tyrosine metabolism are significant only at day 30.

### Weighted correlation network analysis (WGCNA) shows amino acid metabolism enrichment at all radiation time points

We used a complementary approach, weighted correlation network analysis (WGCNA), to investigate correlations between metabolites based on changes in their abundance after radiation. WGCNA does not use information about chemical structure or KEGG pathways, but instead is a completely data-driven method to identify networks of correlated metabolites, the functional modules active in the network, and the “hub” nodes (metabolites in our study) that may drive those biological functions [48]. Thus, WGCNA can incorporate all metabolites from the platform, regardless of whether they have known chemical structures or pathway annotations.

We performed WGCNA analysis in two ways: (1) incorporating all time points together to identify a single set of modules that may change in expression over time, and (2) creating a different network and set of modules for each time point separately. The former can identify groups of metabolites that co-vary over all four stages, whereas the latter can identify groups of metabolites that are only correlated at one specific stage but show unrelated behavior at the other stages (e.g. because they are regulated by an active process during the chronic stage but not at the other stages). After identifying the WGCNA modules, over-representation analysis was performed to obtain pathway enrichment results using KEGG IDs (see S7 Table).

First, we applied WGCNA analysis incorporating all three time points together (S5 Fig and S7 Table). In HILIC phase, we observe significant enrichment of neuroactive ligand receptor interaction (*P*_adj_ <0.05) in the green module (S5A Fig and S7 Table). We note downregulation in the untreated group, upregulation in both the day 3 and day 14 IR groups, and downregulation in the day 30 IR group. The rest of the modules also showed distinct patterns of up and down regulation across different stages, but they were not significantly enriched in any known pathways (S5A Fig and S7 Table). When we applied WGCNA analysis incorporating all three time points together in RP phase, we did not observe significant enrichment of KEGG pathways (*P*_adj_ <0.05) (S5B Fig and S7 Table). As in HILIC phase, the rest of the modules showed unique patterns of up and down regulation across different stages, but they were not significantly enriched in any known pathways (S5B Fig and S7 Table).

When applying WGCNA separately at each stage, we found greater enrichment in KEGG pathways, suggesting that new processes are activated at each stage of salivary gland dysfunction, leading to emergent groups of correlated metabolites. At day 3 IR HILIC phase, 6 metabolite correlation-based modules are identified (S8 Table) and using a *P*_adj_<0.25 cutoff, we observe significant enrichment of purine metabolism (Table 4). The hub, or central metabolite, in purine metabolism is 6-Methoxyquinoline (Table 4). At day 14 IR HILIC phase, 8 metabolite correlation-based modules are detected (S8 Table) and we observe significant enrichment for histidine metabolism and mineral absorption (Table 4). At day 30 IR, 8 modules are identified in HILIC phase (S8 Table) with significant pathway enrichment only observed for arginine biosynthesis (Table 4). At day 3 IR RP phase, 9 modules are identified (S8 Table) and we observe significant enrichment of glyoxylate and dicarboxylate metabolism and purine metabolism with the metabolite hub 3’-Adenosine monophosphate (3’-AMP) (Table 4). At day 14 IR RP phase, 13 modules are identified (S8 Table) and we observe significant enrichment for phenylalanine metabolism and steroid hormone biosynthesis (Table 4). At day 30 IR, 12 modules are identified in RP phase (S8 Table) and no significant enrichment is observed (Table 4). When comparing the top pathway enrichment results for the detection phases across IR time points regardless of statistical significance, phenylalanine metabolism and lysine degradation are common to all IR time points (S8 Table). Glyoxylate and dicarboxylate metabolism is specific to day 3 IR, sphingolipid signaling pathway and thermogenesis are specific to day 14 IR, and ABC transporters and biosynthesis of unsaturated fatty acids are specific to day 30 IR (S8 Table). From the stage-specific WGCNA analysis, we cannot directly conclude whether these modules are up- or down-regulated, only that the upstream signaling that drives these correlations are actively regulating the metabolites in the module.

**Table 4:** Significant (*P*_adj_<0.25) weighted correlation network analysis (WGCNA) metabolite modules with pathway enrichment annotation at days 3, 14, and 30 IR compared to untreated in HILIC and RP phases.

**Day 3 IR HILIC**						
**Module**	**Top module pathway**	***P*adj**	**Pathway size**	**Overlap metabolites**	**Module hub metabolite**	**Hub metabolite name**
green	Purine metabolism—Homo sapiens (human)	0.2043	10	6: Guanine, Adenine, GMP, Hypoxanthine, Adenosine, IMP	C10 H9 N O	6-Methoxyquinoline
**Day 14 IR HILIC**						
**Module**	**Top module pathway**	***P*adj**	**Pathway size**	**Overlap metabolites**	**Module hub metabolite**	**Hub metabolite name**
blue	Histidine metabolism—Homo sapiens (human)	0.0660	9	6: 3-Methylhistidine, L-Aspartate, L-Glutamate, L-Histidinol, Urocanate, L-Histidine	C17 H11 Cl N2 O S	4- [37] benzonitrile
green yellow	Mineral absorption—Homo sapiens (human)	0.1186	11	3: L-Threonine, L-Asparagine, L-Serine	C25 H43 N3 O6	NA
**Day 30 IR HILIC**						
**Module**	**Top module pathway**	***P*adj**	**Pathway size**	**Overlap metabolites**	**Module hub metabolite**	**Hub metabolite name**
red	Arginine biosynthesis—Homo sapiens (human)	0.1441	6	4: L-Aspartate, L-Glutamine, L-Ornithine, N-Acetylornithine	C41 H71 N3 O7	NA
**Day 3 IR RP**						
**Module**	**Top module pathway**	***P*adj**	**Pathway size**	**Overlap metabolites**	**Module hub metabolite**	**Hub metabolite name**
green yellow	Glyoxylate and dicarboxylate metabolism—Homo sapiens (human)	0.0302	6	3: L-Serine, Glycine, 2-Oxoglutarate	C6 H5 N8 O16 P3 S5	NA
cyan	Purine metabolism—Homo sapiens (human)	0.1309	12	7: Guanine, Adenine, Hypoxanthine, Guanosine, 3’-AMP, 5’-AMP, Adenosine	C10 H14 N5 O7 P	3’-Adenosine monophosphate (3’-AMP)
**Day 14 IR RP**						
**Module**	**Top module pathway**	***P*adj**	**Pathway size**	**Overlap metabolites**	**Module hub metabolite**	**Hub metabolite name**
light yellow	Phenylalanine metabolism—Homo sapiens (human)	0.0890	10	3: Benzoate, L-Tyrosine, 2-Phenylacetamide	C30 H56 N4 O3 P2 S	NA
purple	Steroid hormone biosynthesis—Homo sapiens (human)	0.1841	7	3: Pregnenolone, Pregnanediol, Allopregnanolone	C8 H2 Cl N2 O10 P3 S3	NA

No significant enrichment was observed at day 30 IR RP phase. Abbreviations: IR = radiation, HILIC = hydrophilic interaction liquid chromatography, RP = reverse phase chromatography, *P*adj = a Benjamini Hochberg adjusted *P*-value.

### Intersection of amino acids within mitochondrial metabolic pathways

**Fig 4:**
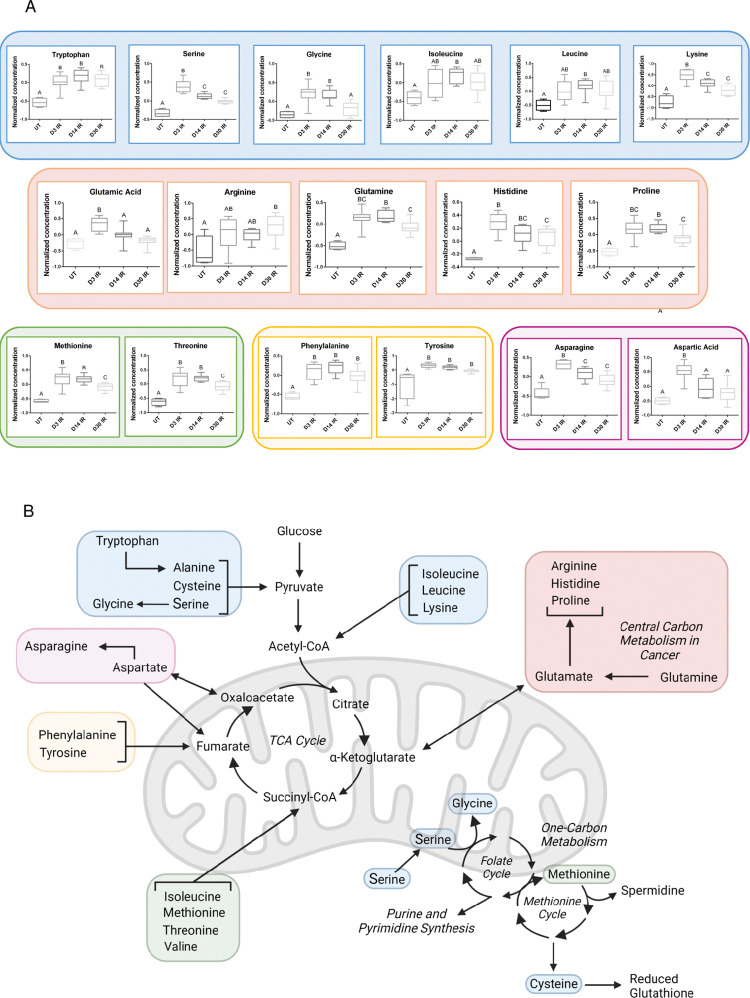
Amino acids connected to mitochondrial metabolism. A) Individual amino acids analyzed by one-way ANOVA with post-hoc Tukey’s test for multiple comparisons. Letters denote statistical significance between groups, *P*<0.05. Box plots display the upper quartile, median, and lower quartile with the maximum and minimum values denoted by the whiskers. N = 4 for untreated (UT), N = 8 for day 3 (D3) IR, N = 8 for day 14 (D14) IR, and N = 8 for day 30 (D30) IR. Alanine, cysteine and valine were not detected in our data set. B) Overview of pathways related to amino acid metabolism. Created in BioRender.com. Abbreviations: *TCA* = tricarboxylic acid.

S9 Table presents a summary of the significant metabolite classes, GSEA pathways and their associated leading-edge metabolites, MetaMapp community pathways, and WGCNA module pathways observed at day 3, day 14, and day 30 IR to compare the findings across the different analytical methods. We observe the highest number of significant metabolites and the highest number of significantly enriched pathways at day 3 IR, and this significance decreases with increasing time as zero significant enriched pathways were observed at day 30 IR from the GSEA analysis. Common enriched pathways across GSEA and MetaMapp network analysis at day 3 and day 14 IR are glutathione metabolism, aminoacyl-tRNA biosynthesis, central carbon metabolism in cancer, and several types of amino acid pathways. Due to the high prevalence of enrichment for amino acid metabolism and significance for amino acids at all IR time points across the three analytical methods (Fig 4), we decided to further investigate amino acid metabolism as an application of this data by interpreting the results in a biological context (see Discussion). Fig 4A displays individual amino acid level changes at each time point in response to radiation, which are further grouped together based on where they interact with mitochondrial metabolic pathways. Fig 4B shows where the grouped amino acids feed into the tricarboxylic acid (TCA) cycle, central carbon metabolism in cancer, and one-carbon metabolism.

## Discussion

This study utilized a metabolomics approach to identify metabolites and pathways altered in the salivary gland in response to IR at acute, intermediate, and chronic damage time points. The results from the current study provide mechanistic insight into the different stages of salivary gland dysfunction following IR. While we identified the greatest number of significantly enriched pathways at the acute damage stage, the metabolites and pathways still altered at the chronic damage time point most likely reflect the pathways of interest to develop targeted interventions against persistent xerostomia. We observed conservation of enriched pathways using different analytical methods across the three time points: glutathione metabolism, aminoacyl-tRNA biosynthesis, central carbon metabolism in cancer, ferroptosis, and various amino acid metabolism pathways. These findings suggest targeting the enriched metabolic pathways conserved across the acute and chronic damage response stages may ameliorate chronic loss of salivary gland function following radiation treatment. Our group’s previous study [24] combined a transcriptomics and metabolomics approach to identify metabolic changes in the salivary gland at day 5 IR, which is when increased compensatory proliferation and decreased apical/basal polarity are observed as the damage state transitions from the acute to the chronic responses (Fig 1) [25, 49]. Our previous study identified coordinated changes in glutathione metabolism, peroxisomal lipid metabolism, bile acid production, and energy metabolism pathways (TCA cycle and thermogenesis) at day 5 IR [24]. In the current study, peroxisomal lipid metabolism, bile acid production, and the TCA cycle and thermogenesis were not identified as significant pathways, and this is most likely due to the MS detection settings not selecting for bile acids and certain TCA cycle intermediates in positive ion mode. Another factor contributing to this discrepancy might be the absence of combinatorial transcriptomic analysis in the present study [24].

In our current study, glutathione metabolism was identified as a significantly enriched pathway at day 3 and day 14 IR using GSEA, and as a significantly enriched pathway at all three IR timepoints using MetaMapp network analysis (S8 Table). Interestingly, reduced glutathione levels were lower in IR vs control at day 5 IR in our previous study [24] while reduced glutathione levels were significantly higher in IR vs control at day 3 in RP phase (log2 fold change (logFC) = 0.902) and at day 14 in HILIC (logFC = 0.976) and RP (logFC = 1.044) phases in the current study (S1 Table). At day 30 IR, reduced glutathione levels were higher (logFC = 0.370) compared to control in RP phase although statistical significance was not reached (S1 Table). A metabolomics analysis of liver tissue in mice exposed to 3 Gy and 11 Gy gamma IR to compare metabolic effects of low versus high dose IR exposure showed significantly higher levels of reduced glutathione levels at both day 4 and day 11 IR compared to untreated tissue, with the highest reduced glutathione levels observed at day 4 compared to day 11 in both the 3 Gy and 11 Gy treatment groups [50], thus supporting the results from our current study. It is well established that glutathione is one of the most prominent radioprotectors in cells as it scavenges free radicals produced from DNA damage [51], which would be a possible explanation for why we observed decreased reduced glutathione at day 5 IR previously as it was utilized to scavenge reactive oxygen species (ROS) that remain elevated in the salivary gland following IR (Fig 1) [31]. The elevation of reduced glutathione observed in the current study and in the metabolomics analysis of IR liver tissue could occur due to the increased synthesis of reduced glutathione from glutamine to fuel protection of the salivary gland tissue from the increase in ROS, which has been previously demonstrated in cancer cells as a protective mechanism against increased oxidative stress [52, 53]. Thus, the mixed results for reduced glutathione observed in our current and previous study may be attributed to flux of the metabolite as it scavenges ROS in the damaged salivary gland tissue over time.

Amino acid metabolic pathways were prominently enriched in the present study across all analytical methods and the three IR timepoints in the GSEA, MetaMapp network modules, and WGCNA module results and include arginine biosynthesis, lysine degradation, histidine metabolism, cysteine and methionine metabolism, phenylalanine, tyrosine and tryptophan biosynthesis, and alanine, aspartate, and glutamate metabolism (S9 Table). We investigated the levels of the detected amino acids that compose these pathways and discovered upon further analysis that all are significantly increased in at least one IR time point compared to untreated (Fig 4A). Multiple studies observed increases in amino acid metabolites in response to IR acutely and chronically. Twelve hours following exposure to total body 6 Gy gamma IR in a mouse model, glutamate and glutamine were increased in the ileum, glutamate was increased in the liver, and phenylalanine in the muscle [54]. At 1, 2, and 3 days following exposure to 2 or 6 Gy x-ray IR in a rat model, phenylalanine was increased in the jejunum, phenylalanine, glutamine, serine, and lysine were elevated in the spleen, and glutamine and serine were increased in the liver [55]. At two months following exposure to 1.6 or 2 Gy gamma IR in a mouse model, histidine, glutamine, and phenylalanine were elevated in intestinal tissue at both IR doses [56]. Thus, it is established that amino acids increase in tissue exposed to IR, but the involvement of these amino acids in various metabolic pathways is not clearly understood.

Amino acid metabolism feeds into many different pathways, several of which were identified as significantly enriched pathways in the current study: central carbon metabolism, glutathione metabolism, and purine metabolism. Central carbon metabolism in cancer refers to various pathways that increase energy production and macromolecule synthesis to sustain tumor growth [57, 58]. There are two main ways that cells accomplish this increase in energy and growth. One method is increasing glucose uptake and the rate of glycolytic flux to increase ATP production quickly, which also increases nucleotide production through the pentose phosphate pathway [57, 58]. A second method is increasing glutamine uptake, which is converted to glutamate and subsequently to other amino acids to synthesize proteins or it feeds into the TCA cycle to increase ATP production [57, 58]. Our previous study identified increased levels of the glutamine transporter transcript *SLC38A1* as well as increased levels of glutamine at day 5 IR in the salivary gland [24]. We observed an increase in glutamine levels at day 3, day 14, and day 30 IR in our current study, and a significant increase in glutamate (glutamic acid) levels at day 3 IR (S1 Table). The leading-edge metabolites in GSEA results for central carbon metabolism in cancer (across all three IR time points) include glutamate, asparagine, aspartate, methionine, phenylalanine, proline, serine, tryptophan, and tyrosine, which suggests increased glutamine levels fueling synthesis of these other amino acids (S4 Table). These results support the hypothesis that IR increases glutamine levels, and subsequently other amino acid levels, potentially to rebuild the damaged salivary gland tissue, similar to what is observed in cancer cells by increasing central carbon metabolism to support tumor growth (Fig 4B). However, the accumulation of amino acids following IR may have a detrimental effect on the function of the salivary gland. Autophagy maintains cellular homeostasis by recycling damaged proteins and is necessary for normal salivary gland function. Morgan-Bathke and colleagues previously demonstrated that administration of the rapalogue CCI-779, an autophagy activator, post-IR treatment significantly improved salivary flow rates at days 30, 60, and 90 post IR compared to mice that only received IR [35]. Therefore, the observed increases in amino acid levels in our current study may be reflective of impaired autophagy in the salivary gland following IR treatment.

Amino acid metabolism is closely linked to glutathione metabolism and purine metabolism through one-carbon metabolism. One-carbon metabolism refers to various anabolic reactions in both the cytoplasm and mitochondria that are responsible for nucleotide synthesis, methylation reactions that affect gene expression, amino acid homeostasis, and reductive/oxidative (redox) defense (Fig 4B) [59, 60]. The methionine cycle is a component of one-carbon metabolism [59], and in our present study we observed significant increases in methionine at all 3 IR timepoints and significant increases in an intermediate in the methionine cycle, S-Adenosylhomocysteine (SAH), at day 3 and day 14 IR (S1 Table). The methionine cycle synthesizes cysteine and reduced glutathione, and our study identified significant increases in reduced glutathione at day 3 and day 14 IR (S1 Table). A leading-edge metabolite annotated to glutathione metabolism, which was significantly enriched in our GSEA output at all IR time points, was spermidine (S4 Table), which is a by-product of the methionine cycle and was increased at all IR time points (Fig 4B), (S1 Table). We observed increases in glycine, serine, and threonine levels at all IR time points in our study, and these three amino acids feed into the folate cycle, which generates purines and pyrimidines (Fig 4A and 4B). Upon further investigation, the detected leading-edge metabolites (S4 Table) for purine metabolism were increased at all IR timepoints (hypoxanthine was the one exception and was slightly downregulated at day 14 IR, although not significantly) and the leading-edge metabolites for pyrimidine metabolism were also increased at all IR time points (S1 Table). Collectively, the increase of cysteine, methionine, glycine, serine and threonine observed in response to IR over time may be linked to the increase in reduced glutathione and purine/pyrimidine levels in the salivary gland through elevated one-carbon metabolism. Previous research has demonstrated that 2, 5, and 7 Gy gamma IR alters one-carbon metabolism in murine liver at days 1, 2, 3, 4, 5, and 8 by shifting priority to nucleotide synthesis at the expense of transmethylation reactions, which can further exacerbate DNA damage [61]. Further investigation of transmethylation products and enzyme levels and activity in the folate and methionine cycles (specifically S-adenosylmethionine) would need to be evaluated to test if the results from our study in the salivary gland support the one-carbon metabolism alteration observed in previous IR research in the liver. Prior research has manipulated the supply of methyl group donors to mitigate the IR response in different model systems, with animal studies displaying significant increases in animal survival, bone marrow health, and intestinal integrity following low- and high-dose IR after receiving glycine betaine as a pre-treatment [62, 63], providing support for further pursuit of this mechanism in IR-induced salivary gland dysfunction as a possible therapeutic target.

A major strength in this study is the use of multiple analytical methods and multiple IR time points, thus increasing the confidence in our identified pathways. To achieve a deeper understanding of the metabolome, multiple analytical techniques are often employed for many reasons. Currently, no single method detects and annotates all features observed in a metabolomics run, but different techniques tend to be more sensitive to different classes of molecule, thereby increasing coverage of the metabolome and sensitivity of the assay. This paid dividends in the MetaMapp analysis and WGCNA, which were more sensitive to HILIC chromatography, while GSEA displayed sensitivity at mapping RP features into pathways. As many of the metabolite annotations were achieved at the MS level, the use of multiple analytical methods also served to validate the results and reduce false positives, increasing confidence in the overall analysis. Using metabolite annotation (GSEA), metabolite structure (MetaMapp), and metabolite levels (WGCNA), we observed conservation of various amino acid metabolism pathways across the different IR time points, which increases the reproducibility of our findings. The network-based analysis in our study aided in creating a biological context for the metabolomics output. The animal model is also a strength as it has previously been demonstrated that FVB mice treated with 5 Gy radiation experience a 40–50% reduction in stimulated salivary flow rates beginning at day 3 and continuing through days 30, 60, and 90 following IR treatment [27]. The use of salivary gland tissue instead of serum, blood, or urine for the metabolomics analysis was a strength as it reduced the influence of metabolites released from other tissues and organs on the metabolomics output. Future studies can use isotope tracing to confirm the directionality of metabolic reactions of interest. This data set is a useful resource for salivary gland biologists investigating mechanisms correlated with radiation-induced disfunction as they can mine the data for metabolic pathways related to their studied mechanism.

An inherent challenge in metabolomics is metabolite annotation. Metabolites vary in structure and lack a common building block, making the synthesis of reference standards for identification challenging despite utilization of several databases including KEGG, HMDB, ChemSpider, or PubChem [64, 65]. Thus, the majority of detected signals in untargeted mass spectrometry runs remain unidentified [66]. Despite the limitations regarding identification, a major strength of this study is the use of an Orbitrap mass spectrometer which allows for sub-ppm confidence in metabolite identification whereas other methods (e.g. linear trap, time-of-flight) only allow for 10–20 ppm confidence [67]. Another limitation of using solely metabolomics analysis is the biological interpretation of the alterations in individual metabolite levels because the metabolites are not identified as reactants or products in a biochemical reaction. For example, increased levels of glutamine could indicate increased output of glutamine, decreased consumption of glutamine, or increased synthesis of glutamine via shunting from another pathway. Due to the complex interplay of metabolic reactions, metabolic flux cannot be simplified to a unidirectional linear model, thus several biological interpretations of the data are possible.

## Conclusions

This study incorporated structural annotation and metabolite level data to identify metabolic pathways altered in the salivary gland at acute, intermediate, and chronic time points following radiation treatment, with the highest number of metabolic changes observed at the acute damage stage. Further investigation of metabolic changes in glutathione metabolism, amino acid metabolism, and central carbon metabolism in cancer may yield promising therapeutic targets to restore loss of tissue function after radiation treatment during the acute damage response to prevent chronic loss of function in the salivary gland.

## Supporting information

S1 Fig PLS-DA performance model for HILIC phase data using cross-validation. R2 reflects the prediction accuracy and Q2 reflects the prediction variation of the groups.
(TIF)

S2 Fig PLS-DA performance model for RP phase data using cross-validation. R2 reflects the prediction accuracy and Q2 reflects the prediction variation of the groups.
(TIF)

S3 Fig MetaMapp network analysis at day 14 IR compared to untreated. A) HILIC phase. B) RP phase. Rectangles correspond to individual metabolites, edges denote chemical reactions between the metabolites, and numbers denote the communities that clusters of metabolites belong to. Color indicates Log_2_ (fold change)—red denotes upregulated metabolite levels versus untreated and blue denotes downregulated metabolite levels versus untreated.
(TIF)

S4 Fig MetaMapp network analysis at day 30 IR compared to untreated. A) HILIC phase. B) RP phase. Rectangles correspond to individual metabolites, edges denote chemical reactions between the metabolites, and numbers denote the communities that clusters of metabolites belong to. Color indicates Log_2_ (fold change)—red denotes upregulated metabolite levels versus untreated and blue denotes downregulated metabolite levels versus untreated.
(TIF)

S5 Fig WGCNA module correlation to condition combining untreated (D0), day 3 IR (D3), day 14 IR (D14), and day 30 IR (D30) groups. Each module shows the correlation as the top number and the corresponding p-value as the bottom number. Red denotes upregulation and blue downregulation. A) HILIC phase. B) RP phase.
(TIF)

S1 Table Full list of metabolites obtained from days 3, 14, and 30 IR mouse parotid gland samples (N = 8/group) using ultra-high performance liquid chromatography-mass spectrometry and processed using Compound Discoverer software in HILIC and RP phases. (XLSX)

S2 Table Top 100 significant metabolites (*P*_adj_<0.05) with metabolite class annotation at days 3, 14, and 30 IR in HILIC and RP phases. (XLSX)

S3 Table Full GSEA pathway enrichment results at days 3, 14, and 30 IR in HILIC and RP phases. (XLSX)

S4 Table Leading-edge metabolites in enriched pathways at days 3, 14, and 30 IR in HILIC and RP phases. (XLSX)

S5 Table Full list of identified MetaMapp metabolite communities at days 3, 14, and 30 IR in HILIC and RP phases. (XLSX)

S6 Table Full list of pathways annotated to MetaMapp metabolite communities at days 3, 14, and 30 IR in HILIC and RP phases. (XLSX)

S7 Table Pathway enrichment results for weighted correlation network analysis metabolite modules combining untreated, days 3, 14, and 30 IR in HILIC and RP phases. (XLSX)

S8 Table Pathway enrichment results for individual weighted correlation network analysis metabolite modules at days 3, 14, and 30 IR in HILIC and RP phases. (XLSX)

S9 Table Summary of significant metabolites and pathways from each analysis describing the metabolic response to radiation over time in the salivary gland. (XLSX)
