# Supplementary figures and images for: Metabolomics analysis of pathways underlying radiation-induced salivary gland dysfunction stages
Source: PLoS One. 2023 Nov 20;18(11):e0294355. doi: 10.1371/journal.pone.0294355 (PMC10659204; doi:10.1371/journal.pone.0294355)

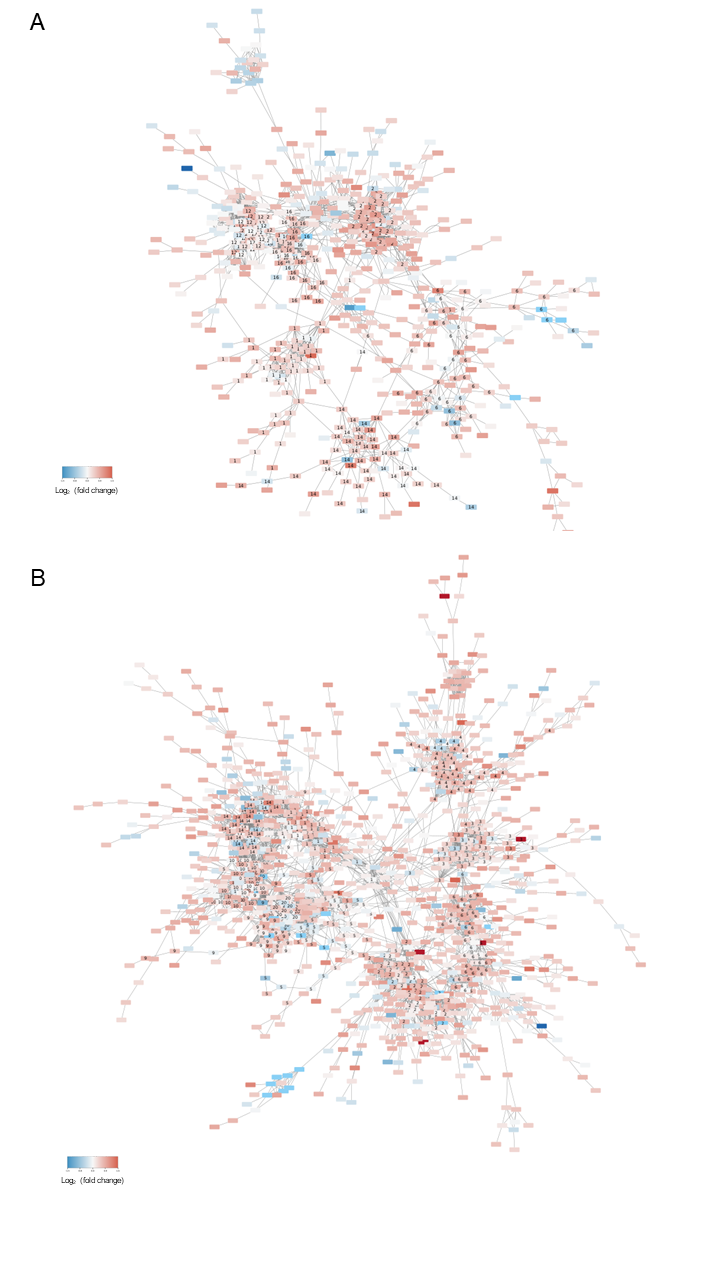

Supplement: S1 Fig — R2 reflects the prediction accuracy and Q2 reflects the prediction variation of the groups. (TIF) [file pone.0294355.s001.tif]

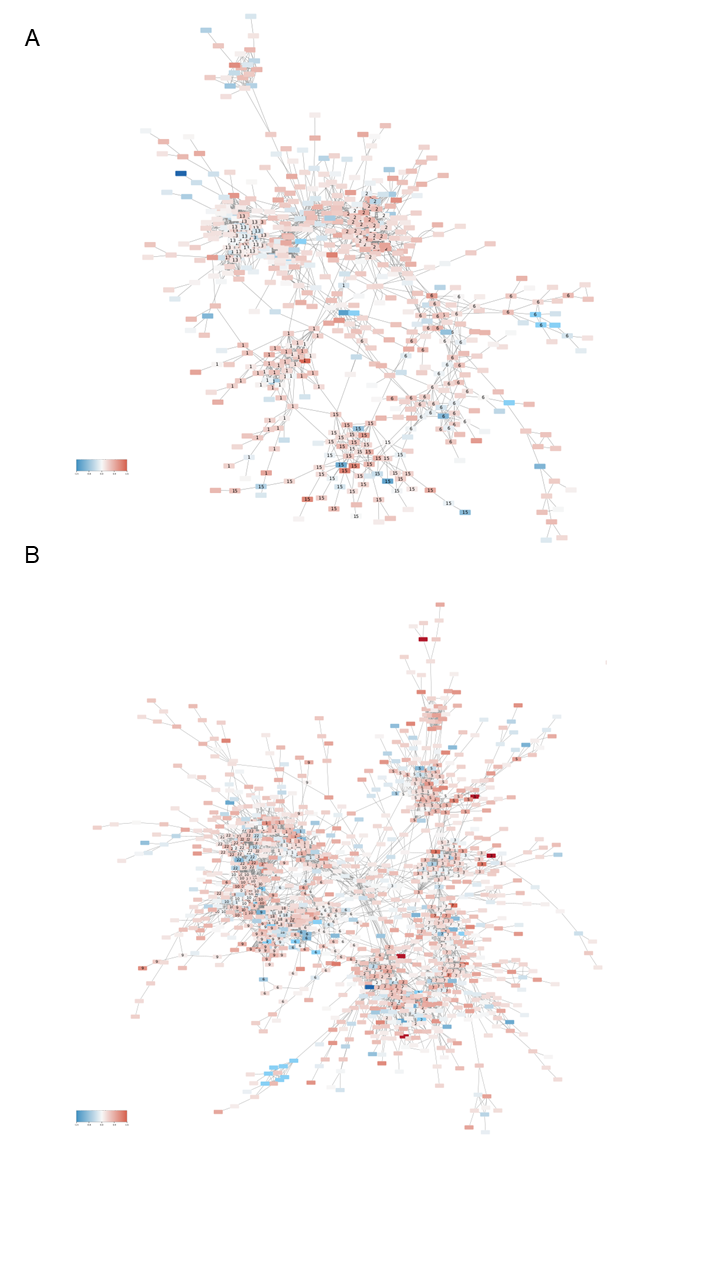

Supplement: S2 Fig — R2 reflects the prediction accuracy and Q2 reflects the prediction variation of the groups. (TIF) [file pone.0294355.s002.tif]

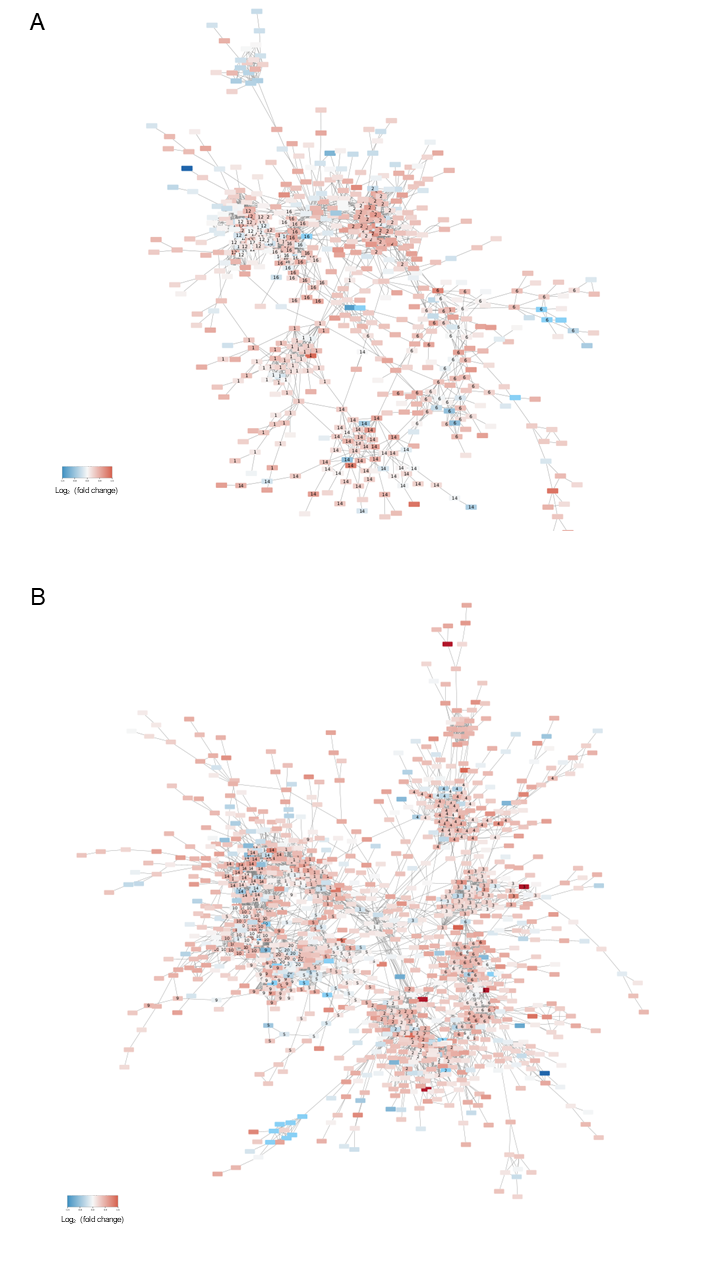

Supplement: S3 Fig — A) HILIC phase. B) RP phase. Rectangles correspond to individual metabolites, edges denote chemical reactions between the metabolites, and numbers denote the communities that clusters of metabolites belong to. Color indicates Log2 (fold change)—red denotes upregulated metabolite levels versus untreated and blue denotes downregulated metabolite levels versus untreated. (TIF) [file pone.0294355.s003.tif]

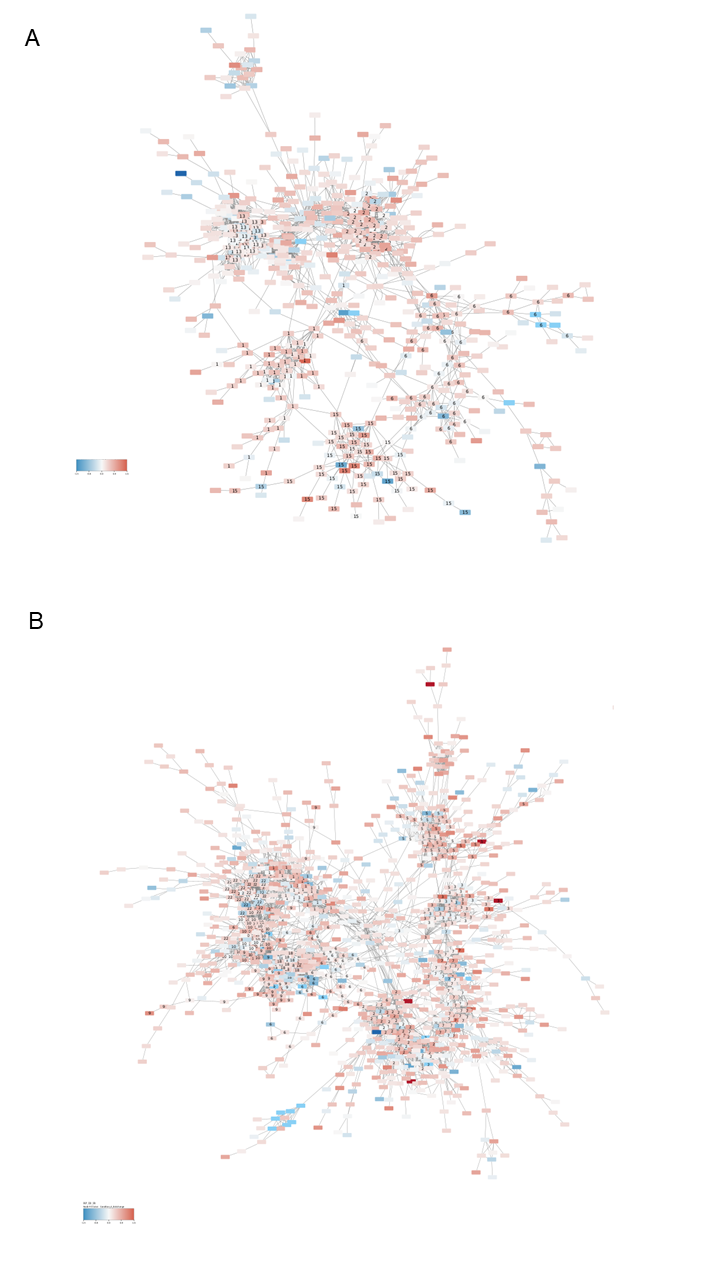

Supplement: S4 Fig — A) HILIC phase. B) RP phase. Rectangles correspond to individual metabolites, edges denote chemical reactions between the metabolites, and numbers denote the communities that clusters of metabolites belong to. Color indicates Log2 (fold change)—red denotes upregulated metabolite levels versus untreated and blue denotes downregulated metabolite levels versus untreated. (TIF) [file pone.0294355.s004.tif]

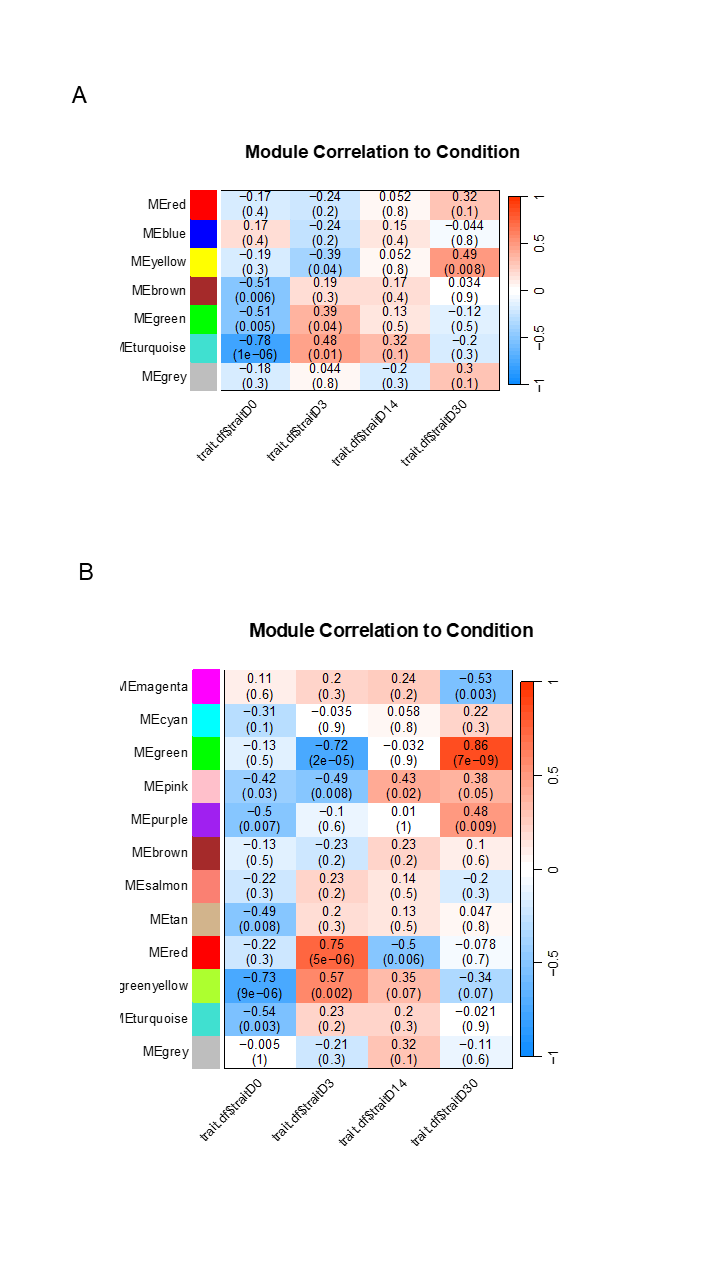

Supplement: S5 Fig — Each module shows the correlation as the top number and the corresponding p-value as the bottom number. Red denotes upregulation and blue downregulation. A) HILIC phase. B) RP phase. (TIF) [file pone.0294355.s005.tif]
